# Raman Spectroscopy and Surface Enhanced Raman Scattering (SERS) for the Analysis of Blue and Black Writing Inks: Identification of Dye Content and Degradation Processes

**DOI:** 10.3389/fchem.2019.00727

**Published:** 2019-10-25

**Authors:** Daniela Saviello, Maddalena Trabace, Abeer Alyami, Antonio Mirabile, Piero Baglioni, Rodorico Giorgi, Daniela Iacopino

**Affiliations:** ^1^Nanotechnology Group, Tyndall National Institute, University College Cork, Cork, Ireland; ^2^Department of Chemistry & CSGI, University of Florence, Florence, Italy; ^3^Mirabile, Paris, France

**Keywords:** ballpoint pens, dyes, Raman, plasmonic nanopastes, SERS, degradation

## Abstract

Raman spectroscopy and Surface Enhanced Raman Scattering (SERS) were applied to the analysis of blue and black writing inks. SERS was performed by application of plasmonic nanopastes constituted by Ag nanoparticles and Au nanorods directly on inks deposited on paper substrates under laser irradiation of 514 nm. It was found that SERS spectra were largely enhanced compared to Raman spectra and that Ag nanopastes produced much larger enhancements than Au nanopastes, due to a combination of plasmonic resonance, charge transfer, and molecular resonance effects. All analyzed writing inks resulted constituted by Crystal Violet and other triarylmethane dye mixtures, to which sometimes phthalocyanine dyes were also added (for example in Bic pens). SERS was also used for the identification of degradation processes occurring in artificially aged blue pens deposited on paper substrates. It was found that color alteration changed from ink to ink and varied from darkening to discoloration to slight fading, depending on the initial chemical composition. For inks containing Crystal Violet, two mechanisms associated to de-methylation and photo-reduction of excited dye to colorless leuco forms were identified.

## Introduction

Modern commercial inks are a complex mixture of several dyes and/or pigments constituting up to the 50% of the total ink formulation, carried either in a glycol-based solvent or benzyl alcohol (Bell, 2008; Houck and Siegel, 2010; Houck et al., 2013). Analysis of commercial writing inks is usually carried out in the context of forensic examination in order to assess dating and originality of documents and to correctly assess cases of crimes like falsification, questioned signatures and threatening letters. Accordingly, a vast number of publications in the last 20 years have focused on characterization of writing pens (i.e., identification of dyes and pigment contents in ballpoint and gel pens) and also on discrimination between inks. Therefore a number of techniques such as Thin Layer Chromatography (TLC) and mass spectrometry (MS) often coupled with optical analysis (ink absorption and luminescence) or image analysis have been routinely applied to the analysis, differentiation, and classification of writing inks (Weyermann et al., 2007; Djozan et al., 2008; Gallidabino et al., 2011; Sauzier et al., 2015). Recently, visible, Raman, and infrared spectroscopies have also gained popularity as techniques for providing rapid information on the chemical content of the ink mixtures and are increasingly pursued for their rapidity of analysis, little sample preparation and non-destructive analysis. Such approaches are also particularly amenable to the investigation of ink-based paper works, whereby the identification of dyes and pigment ink constituents is important to assess dating, originality and to implement correct conservation procedures thus ensuring long-term preservation. Among spectroscopic methods, Raman and Surface Enhanced Raman Scattering (SERS) spectroscopies have been widely used for identification of colorants in artworks and have shown as promising techniques for the identification of dye components in commercial inks (Mazzella and Buzzini, 2005; Bráz et al., 2014; Calcerrada and García-Ruiz, 2015). In particular, SERS revealed very effective in quenching the interference strong fluorescence emission sometimes accompanying the direct Raman illumination of writing inks thus enabling generation of high intensity spectra even under visible laser irradiation (Jeanmaire and Van Duyne, 1977; Geiman et al., 2009; Luo et al., 2013). However, the majority of SERS results suffer from poor reproducibility arising from the use of randomly aggregated Ag colloidal solutions, usually dropped on extracted samples (Seifar et al., 2001; White, 2003). In contrast, in order to guarantee reproducibility of analysis, SERS requires the use of reliable and robust plasmonic probes ideally applicable directly on the paper substrate. Recently, our group has developed plasmonic probes comprising Ag and Au nanopastes and nanoinks and has used SERS in combination with UV-vis spectroscopy for the analysis of blue and colored Bic pens, increasingly used for artistic purposes (Alyami et al., 2016, 2017, 2018).

Another important consideration in the field of artwork preservation is the aging of the ink deposited on a substrate (usually, but not necessary, paper). As soon as an ink line is deposited on a substrate, compositional changes occur rapidly due to loss of volatile components, photodegradation of dyes or polymerization of resins. All these processes happen concomitantly and they all contribute to significantly alter the aesthetic of paper works exposed to environmental conditions. Photofading of ballpoint pens has been traditionally performed by laser desorption/ionization (LDI) and matrix-assisted laser desorption/ionization (MALDI) mass spectrometry (Weyermann et al., 2006). However, in light of the need to preserve the integrity of analyzed objects, non-destructive techniques are nowadays preferred for ink-based artworks. Accordingly, Raman spectroscopy has been successfully used for the investigation of dye aging dynamics in writing inks and SERS was successfully applied by Cesaratto et al. to the tracking of triarylmethane dyes photodegradation on nineteenth century woodblock Japanese prints (Gorshova et al., 2016; Cesaratto et al., 2017).

In this work we propose the use of Raman and SERS spectroscopy for the analysis of a number of blue and black writing inks on paper substrates. The aim of the present study is to evaluate the ability and limitations of Raman spectroscopy for the analysis of writing inks and to assess the improvements brought about by the use of SERS. This includes routine chemical make-up identification as well as more complex analysis such as degradation assessments. The ability of SERS in overcoming fluorescence background limitations and increasing the sensitivity of Raman spectroscopy have been investigated already. However, results are contradictory as robust SERS probes for such type of analysis are missing. The novelty of the presented manuscript relies on the development of SERS metal nanopastes providing a reproducible method for the investigation of signal enhancement effects associated with the use of SERS compared to Raman spectroscopy. This is relevant for analysis of artworks whereby only small size samples are available for analysis. Moreover, the ability to synthesize SERS nanopastes of different plasmonic properties (Ag, Au) widens the application of SERS spectroscopy to Raman instrumentations of different excitation wavelengths and also to portable/handheld instrumentation.

## Materials and Methods

### Materials

Tetrachloroauric acid (99.5%), silver nitrate (ACS, 99%), sodium citrate (99 %), sodium borohydride (96%), ascorbic acid (CAS: 50817), cetyltrimethylammoniumbromide (CTAB, 99%), MeOH (CAS: 67561) were purchased from Sigma-Aldrich. All glassware was cleaned with *aqua regia* prior to nanopaste synthesis. Milli-Q water (resistivity > 18 MΩ cm^−1^) was used throughout the experiments. Reference dye Crystal Violet (CAS: 548629) was also purchased from Sigma and used without further purification.

### Synthesis of Plasmonic Nanopastes

Silver nanoparticle nanopastes were synthesized following the modification of the Lee and Meisel method reported by Polavarapu et al. (2014). Briefly, trisodium citrate solution (4.5 mL, 1.00 wt%) was added to an aqueous boiling solution containing AgNO_3_ (200 mL, 42 mg) under vigorous stirring. The reaction was boiled for another 1 h and then cooled to room temperature. The obtained Ag nanoparticles in water (200 mL) were centrifuged at 7,000 rpm for 20 min and then re-dispersed in water (2 mL) to obtain Ag nanopaste (3 mg/mL). Au nanorod nanopastes were synthesized by a modification of the seed mediated growth reported by Polavarapu et al. (2014). Specifically, a seed solution was prepared by adding 0.3 mL of an ice-cold aqueous sodium borohydride (NaBH_4_, 0.01 M) solution to an aqueous solution of 4.7 mL hexadecyltrimethylammonium bromide (CTAB, 0.1 M) and 25 μL of gold(III) chloride trihydrate (HAuCl_4_, 0.05 M) at 30°C. An aliquot of 0.36 mL of the seed solution was added to a growth solution prepared by mixing 150 mL of CTAB (0.05 M), 1.5 mL HAuCl_4_ (0.05 M), 0.225 mL of silver nitrate (AgNO_3_, 0.01 M), and 5.0 mL of ascorbic acid (0.1 M) at 30°C. The solution color changed from colorless to brownish-bluish after the addition of the seed solution to the grown solution. The obtained aqueous solution of gold nanorods was centrifuged twice and re-dispersed in water (1 mL) to get the nanorod paste.

### Commercial Writing Pens

Commercial writing pens were purchased from local stores. Real pen artworks were made by French artist Anne-Flore Cabanis and were kindly donated by paper conservator Antonio Mirabile. Artworks were made by Bic blue and black pens on commercial paper.

### Scanning Electron Microscopy

Scanning electron microscopy (SEM) images of nanoinks deposited on SiO_2_ substrates were acquired using a field emission SEM (JSM-6700F, JEOL UK Ltd.) operating at beam voltages of 2 kV.

### Thin Layer Chromatography

Thin layer chromatography was performed with silica plates 10 × 20 cm (Sigma-Aldrich). The writing inks and reference dye Crystal Violet were deposited as concentrated MeOH solutions. The TLC was developed in ethylacetate-ethanol-water (70:35:30 v/v) for 60 min.

### Optical Characterization

UV-Vis spectra were acquired with an Agilent/HP 8453 UV-Vis Spectrophotometer (200 nm < λ < 1,100 nm). Raman spectra at 514 nm were obtained from a Renishaw inVia Raman system. An argon ion laser (1,800 l/mm grating) was employed as an excitation source. The laser beam was focused onto the sample through a Leica 20X objective with 0.4 N.A. Measured power at the sampling level was controlled at about 3 mW. Acquisition time was usually 10 s. Raman spectra at 785 nm were obtained from a Pelkin Elmer Raman station. The laser beam was focused onto the sample through a 50X objective (M Plan Achromat) with 0.75 N.A. The laser power was around 3.5 mW and typical acquisition time was 10 s. To obtain SERS spectra, 5 μL of plasmonic nanopastes were deposited on pen written lines on paper and left to evaporate for 30 min prior analysis.

### Artificial Aging

Paper samples with written blue pen features were subjected to three artificial aging cycles as follows: thermo-hygrometric and photo-oxidative for a total duration of 88 days. Every cycle comprised three phases: an initial phase in humidity chamber with thermo-hygrometric control of temperature at 25.5°C and 80% relative humidity; a second phase of thermal degradation in oven at 80°C and a third phase of neon light exposure to the front and back of the paper sheet.

## Results and Discussion

### Metal Nanopastes SERS Probes

Writing inks from 10 commercial pens (eight blue and two black) were analyzed. Details of used pens and optical microscopy images of deposited inks on paper are reported in Figure S1.

**Figure 1:**
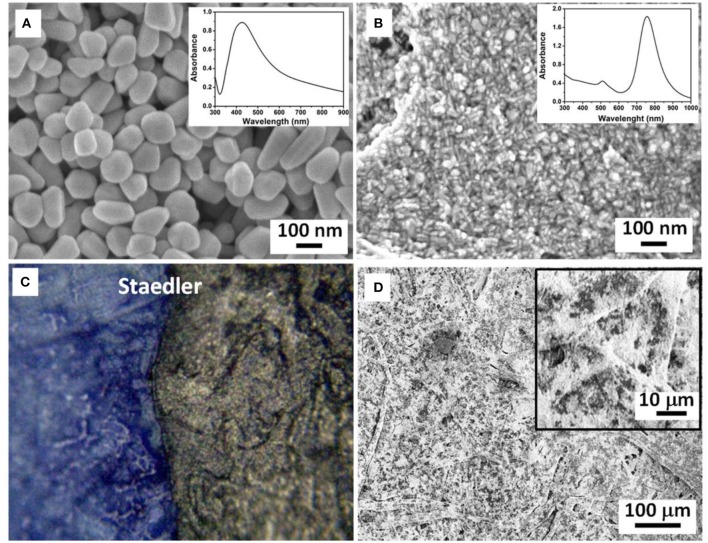
SEM images of **(A)** Ag nanoparticle and **(B)** Au nanorod nanopaste deposited on SiO_2_ support. Insets: UV-vis spectra of Ag nanoparticle and Au nanorod nanopaste in aqueous solution; **(C)** optical microscopy image of blue Stadler pen on paper with deposited droplet of Ag nanopaste; **(D)** SEM image of Ag nanopaste droplet on Stadler colored paper. Inset: high magnification SEM image showing even distribution of paste on the paper fibers.

**Figure 2:**
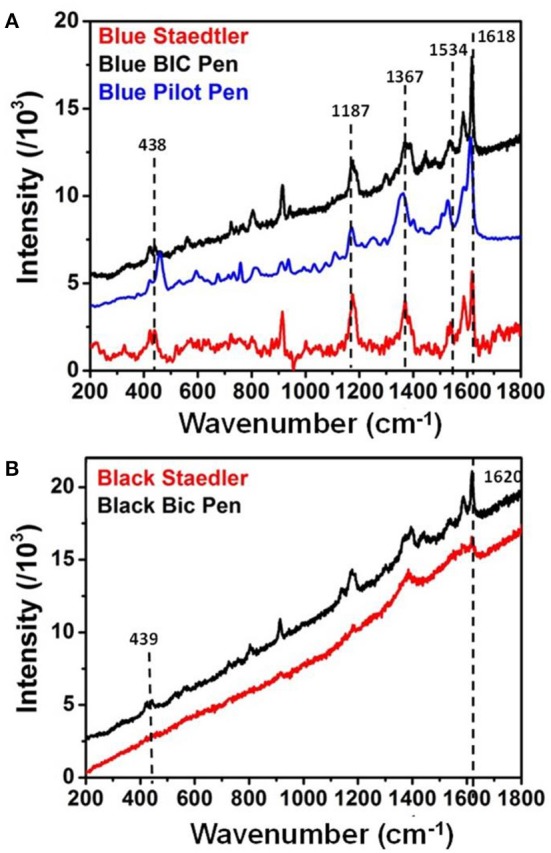
Raman spectra of **(A)** blue Bic, Staedler, and Pilot inks on paper and **(B)** black Bic, Staedler inks on paper. Spectra were taken with 514 nm illumination wavelength.

In order to perform SERS analysis directly on the pen lines, and therefore to avoid lengthy extraction processes, it is necessary to use colloidal plasmonic solutions able to homogeneously cover the colored paper fibers, in order to ensure reproducibility of results and high density of hot spots necessary for generation of high intensity spectral features. Toward this end, Ag nanoparticle and Au nanorod nanopastes were synthesized by slight modification of the “pen-on-paper” method proposed by Polavarapu et al. (2014). Figures 1A,B show SEM images of the synthesized nanopastes deposited on n-doped conductive SiO_2_ substrates. Ag nanopastes were mostly spherical in shape and had an average size of 65 ± 3 nm; the Au nanopastes displayed an elongated (rod-like) shape with average size of 13 ± 2 × 41 ± 4 nm. The absorbance spectra of synthesized nanopastes are reported as insets: the Ag nanopaste solution showed a relatively large plasmonic peak centered at 426 nm whereas the Au nanopaste solution was characterized by two peaks at 520 and 787 nm, associated to transversal and longitudinal plasmon resonances occurring in elongated nanoparticles (Johnson et al., 2002). Figure 2B shows a representative optical microscopy image of deposited Ag nanopaste on blue Staedler pen, showing formation of a well-defined SERS-active area on the analytical surface without smudging nor dissolution of the pen ink underneath. The representative SEM image showed in Figure 1C shows highly homogeneous distribution of plasmonic nanoparticles over large areas (>0.6 mm^2^) of the colored paper fibers. Such high density of adsorbed Ag nanoparticles results in a high density of hot spots suitable for generation of high intensity SERS spectra.

### Raman Spectroscopy and SERS of Writing Inks

Prior measurements of SERS spectra Raman spectra were recorded for five pens, in order to establish limitations of Raman spectroscopy and assess enhancement generated by subsequent application of SERS. All Raman spectra were recorded with 514 nm excitation wavelength and 3 mW laser power and are shown in Figure 2 without background subtraction. Raman spectra of blue and black Bic and blue Staedler pens (Figures 2A,B) showed very similar bands (see Table 1 for details) at 1,618, 1,587, 1,540 cm^−1^, which were associated to the stretching of the benzene rings in triarylmethane compounds and 439, 420 cm^−1^ bands associated to bending of CNC bonds (Canamares et al., 2008). Based on previous findings by our group and others, the presence of crystal violet (CV, basic violet 3, C. I. 42555) was hypothesized for these pen inks (Alyami et al., 2016, 2017). It should be noted that previous analysis carried by our group on blue Bic pen ink whose components were separated by thin-layer chromatography (TLC) and subsequently identified by Raman/SERS spectroscopies showed also presence of a blue phthalocyanine component in the ink formulation. However, the phthalocyanine component did not give any Raman response at 514 nm excitation, confirming the results obtained in this work on the overall Bic pen ink showing only contribution from the CV component (Kunicki et al., 2013; Alyami et al., 2017). The Raman spectrum of blue Pilot pen (Figure 2A) showed good intensity bands centered at 1,614, 1,530, 1,359, 1,168, 438, and 419 cm^−1^, which could also be attributed to the presence of a triarylmethane mixture but not specifically to CV. The Raman spectrum of black Staedler (Figure 2B) also indicated possible presence of CV, although the intensity of obtained Raman band was too low and affected by significant fluorescence interference to allow unequivocal peak assignment.

**Table 1:** List of band positions found in Raman spectra of analyzed inks.

**Blue Bic**		**Blue staedler**	**Blue pilot**	**Black Bic**	**Black stadler**	Assignments^*^
1,618	A1	1,618	1,614	1,620	1,620	
1,583	E	1,587	1,583	1,585	1,584	
1,534	E	1,540	1,528	1,532		ν(C_ring_N)δ_s_(CH_3_)
1,387	E			1,395		δ(CH)/δ_s_(CH_3_)/δ(CCC)_ring_
1,367	A1	1,369	1,359		1,382	ν_as_(CC_center_C)/δ(CCC)_ring_/δ(CH)
1,173	A1	1,170	1,168	1,174		ν_s_(CC_center_C)/δ(CCC)_breathing_/ρ_r_(CH_3_)
913	E	914	911			δ(CC_center_C)
438	A1	440	438			δ(CNC)
421	E	423	419	420		δ(CNC)/δ(CC_center_C)

* *ν, stretching (s, symmetric; as, asymmetric); δ, bending*.

**Figure 3:**
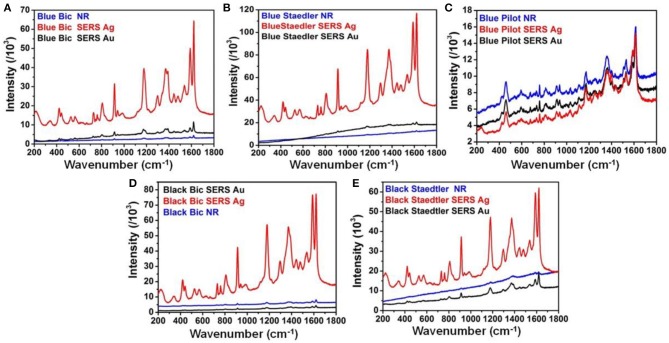
Comparison between Raman and SERS spectra for **(A)** blue Bic pen; **(B)** blue Staedler pen; **(C)** blue Pilot pen; **(D)** black Bic pen; and **(E)** black Staeadler pen. All spectra were taken with 514 nm illumination wavelength and laser power 3 mW.

Figure 3 show SERS spectra recorded for each analyzed pen with both Ag and Au nanopastes. SERS spectra of additional blue writing inks are reported in Figure S3. The SERS spectra of each pen were plotted together with the corresponding Raman spectrum in order to give a straight forward visualization of the degree of enhancement obtained with both SERS probes compared to Raman conditions. In addition the blank Raman and SERS spectra of commercial Fabriano paper were also taken and are shown in Figure S2. The blank Raman spectrum was featureless; low intensity and broad peaks at 1,595, 1,519, and 1,407 cm^−1^ where observed with Ag nanopastes, one peak at 1,619 cm^−1^ peak was observed with Au nanopastes. The application of Ag nanopastes resulted in generation of high intensity SERS spectra for blue and black Bic and Staedler pens (Figures 3A–E). Specifically, spectra displayed intensities ca. 1 order of magnitude higher than intensities of corresponding Raman spectra (measured using the intensity of the 1,180 cm^−1^ band as reference) and showed all diagnostic peaks of CV. This enhancement was mainly attributed to an electromagnetic effect (EM), due to the use of an illumination wavelength in plasmonic resonance with the Ag nanopastes (Figure 1C). Details of the position of the bands found in all analyzed inks are reported in the SI (Table S1). The high intensity and clear formation of diagnostic bands allowed unequivocal identification of CV in both Bic and Staedler blue and black pen inks. Closer observation of the Bic and Staedler blue and black SERS spectra showed a red shift of frequencies compared to the Raman spectra. This in turn was associated to a chemical effect (CE) due to the formation of a chemical bond, and consequent charge transfer (CT), between CV and Ag via the central C atom whereby CV acted as an electron donor and Ag as electron acceptor (Canamares et al., 2008). Specifically, the C = C ring frequency was shifted from 1,618 to 1,620 cm^−1^ for the above ink pens. Further proof of occurrence of a CE was found in the following observed shifts: for Bic blue the N-phenyl frequencies appearing at 1,367 and 1,387 cm^−1^ in the Raman spectrum were shifted to 1,371 and 1,392 cm^−1^, respectively in the SERS spectrum; for blue Staedler shifts from 1,369 and 1,386 cm^−1^ to 1,371 and 1,389 cm^−1^ were measured; for Bic/Staedler black inks bands were located at 1,370/1,373 and 1,388/1,388 cm^−1^, but resolution of the Raman spectrum was too low to measure bands positions, respectively. Further proof of the occurrence of a CT process was given by the predominant enhancement of the non-totally symmetric (E) modes compared to the totally symmetric (A1) modes (Canamares et al., 2008; Selvakannan et al., 2013). As well as a CE a molecular resonance (MR) effect occurred for these pen inks, being the excitation wavelength in resonance with the absorption maximum of CV (588 nm with shoulder at 548 nm). Therefore, the large enhancement observed was attributed to the joint play of three effects: MR, EM, and CE.

In contrast, much less pronounced enhancement was obtained with Au nanopastes. This was expected as the major longitudinal plasmonic band of Au nanopastes was centered at 787 nm, away from the SERS excitation wavelength. The small enhancement observed could be attributed to a modest electromagnetic effect associated to the transversal band at 520 nm and to the ability of CV to form a CT complex with Au via the N groups (Selvakannan et al., 2013).

Presence of phthalocyanine in blue Bic pen was confirmed by SERS spectrum taken at 785 illumination wavelength with Au nanopastes (Figure S4a). In contrast SERS spectrum of blue Staedler taken at 785 nm with Au nanopastes (Figure S4b) showed bands in same positions as SERS bands recorded at 514 nm illumination, therefore proving no presence of additional dye components. Surprisingly, the application of both Ag and Au nanopastes resulted in no enhancement generation for the blue Pilot pen ink (Figure 3C), suggesting a marked different chemical composition compared to the Bic and Staedler ink mixtures. The presence of Victoria blue R component was hypothesized for this pen ink based on resemblance between the recorded spectrum at 785 nm (see Figure S4c) with the spectra of Pilot pens and reference dyes reported in literature (Ho et al., 2016). Specifically, bands at 1,611, 1,360, 1,178, 760, 732, 460, and 421 cm^−1^ overlapped the spectrum of Victoria R reference taken at 785 nm reported in literature.

### Raman Spectroscopy of Real Artworks

**Figure 4:**
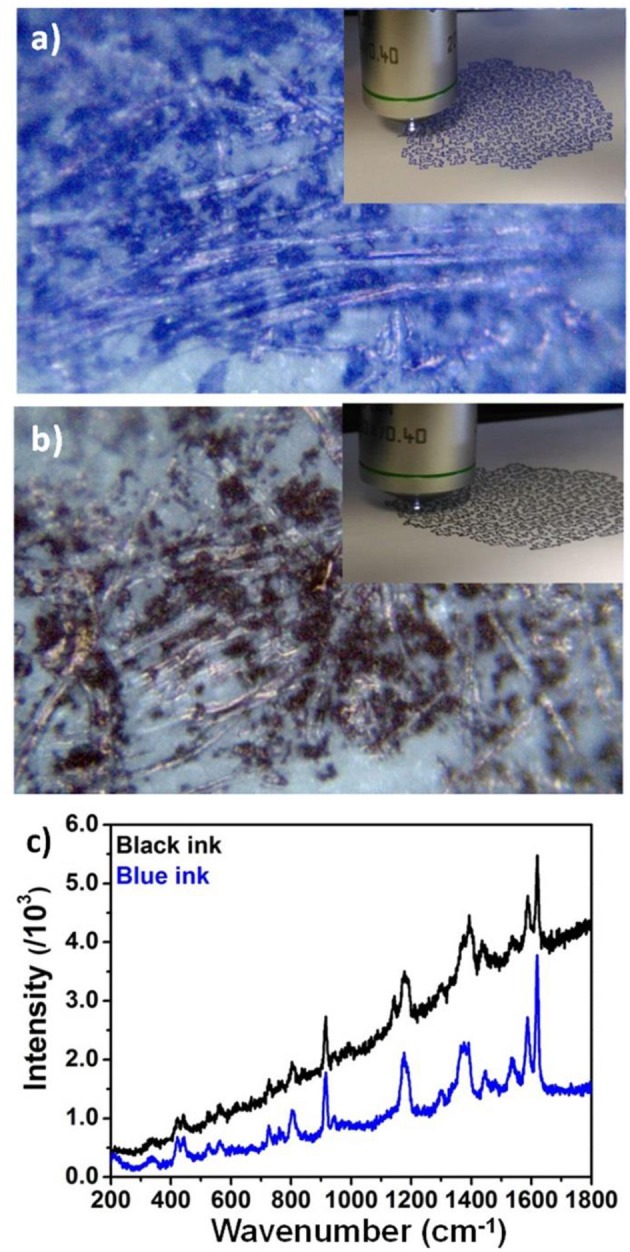
Optical microscope images of artistic drawings realized with **(a,b)** blue pens on paper. Insets: photographs of the entire drawings under the Raman spectrograph objective; **(c)** Raman spectra of the artistic drawings shown in **(a,b)**.

In order to show the importance of database spectral collection for identification of inks used in artworks Figure 4 shows Raman analysis performed on two artistic contemporary drawings made by writing blue and black pens. Specifically, Figures 4a,b shows optical microscopy images of the pen lines on the paper with areas of dense inks spots dis homogeneously distributed on the paper substrate, due to the dis homogeneous and fibrous morphology of the paper itself. Figure 4b shows Raman spectra of the blue and black drawings. Both spectra show bands associated to CV at 1,618, 1,586, 1,532, 1,445, 1,172, 916, 441, and 403 cm^−1^. Careful comparison with Raman spectra of Bic pen (Figure 2) strongly suggested that this pen was used for the realization of the artistic drawing. As well as overlapping peak positions, the ratio of peaks 1,620/1,588 = 1.75 is equal to the ratio found in blue and black BIC pen Raman spectra.

### SERS for Investigation of Writing Inks Aging Processes

As writing inks are increasingly used for the realization of drawings and mixed media artistic creations it is of paramount importance to carry out investigations on color changes brought upon exposure of ink-based objects to light as such exposure is known to induce severe color changes compromising the aesthetic, informational, and value of such objects. Moreover, the elucidation of mechanisms leading to color changes in ink dyes is important for characterization and identification of documents and objects, identification of ink-dye constituents and informative for the application of tailored conservation and exposure protocols.

**Figure 5:**
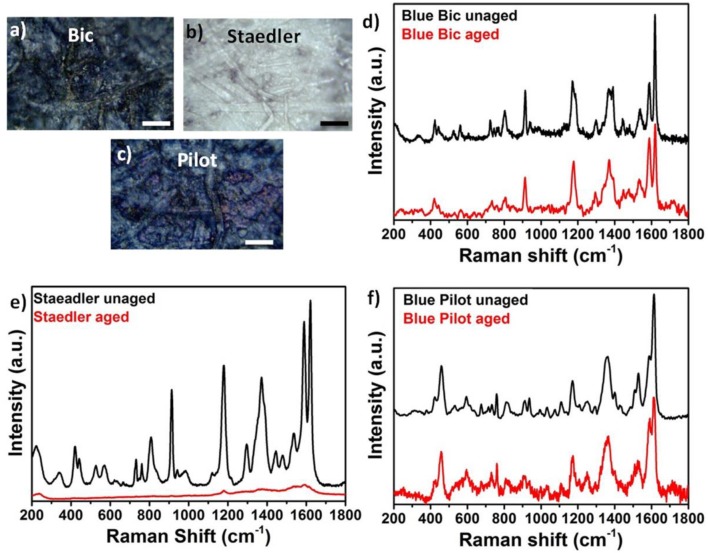
**(a–c)** Optical microscopy images of artificially aged blue Bic, Staedler, and Pilot inks on paper; **(d–f)** Comparison between aged and unaged SERS spectra for blue Bic, Staedler, and Pilot inks on paper.

Figures 5a,c,e show optical images of blue Bic, Staedler, and Pilot inks on paper taken after three cycles of artificial aging. Compared to unaged conditions minor color changes were observed for the Pilot pen whereas Bic and Staedler pens showed substantial color changes resulting in darkening of the Bic pen ink and a dramatic discoloration of the Staedler pen ink. Corresponding SERS spectra of aged inks are reported in Figures 5b,d,f. Spectra were obtained with Ag nanopaste and measured at 514 nm laser excitation wavelength. For easiness of comparison aged pen SERS spectra were plotted together as the equivalent SERS spectra (i.e., same laser power and exposure) of unaged pen inks. Spectra were background subtracted in order to allow more direct comparison of peak intensity changes. The SERS spectrum of aged Bic pen showed diagnostic bands of CV. Compared to the unaged SERS spectrum, few key changes were observed. The ratio I_1620_/I_1587_ decreased from the 2.21 value in the unaged sample to the 1.19 value in the aged samples, the CV peak at 723 cm^−1^ shifted to 732 cm^−1^; the peaks at 744 and 771 cm^−1^ disappeared and the shoulders at 1,189 and 1,390 cm^−1^ disappeared. All these changes are in agreement with a mechanism of photo-induced N-demethylation already observed by Cesaratto et al. during their artificial aging studies on triarylmethylene dyes (Cesaratto et al., 2017). Specifically, the spectral changes observed in the Bic pen corresponded to what Cesaratto et al. observed at step 2 illumination, corresponding to a light dose of 0.12 MIx·h and associated to the occurrence of a N-demethylation process. Interestingly, faster degradation was observed for Staedler pen, as represented by the strong discoloration of exposed paper. Broad peaks were observed in the SERS spectrum of the aged pen centered at 1,613, 1,555, 1,536, 1,178, and 909 cm^−1^.

Comparison of spectra showed strong resemblance with step 4 illumination (2.10 MIx·h) described by Cesaratto et al. and indicative of a chemical conversion from CV to fully *N*-demethylated pararosaniline. These results are in agreement with data reported by Weyermann et al. who found that degradation kinetics of inks deposited on paper differed substantially from pen to pen (Weyermann et al., 2006). A few spectral changes also occurred for Pilot pen, although the uncertain dye mixture identification did not allow correlation with any specific degradation process. Specifically, peaks at 533, 937, and 1,073 cm^−1^ disappeared in the aged pen; also and the ratio I_1616_/I_1587_ decreased from 1.97 to 1.29 following the artificial aging process.

The striking color difference between aged Bic and Staedler pens was ascribed to the chemical composition of their constituting ink. It is known that Bic ink formulations contain a mixture of blue phthalocyanine and triarylmethane dyes and that while the latter are extremely sensitive to photo- and thermal irradiation, phthalocyanine dyes degrade at a much lower rate compared to triarylmethane dyes. Interestingly and in contrast with common perception that aging induces color fading, our optical images showed a darker blue coloration in aged Bic pen inks, probably due to residual presence of phthalocyanine dye. In contrast, strong discoloration was observed in the Staedler pen, which further proves lack of phthalocyanine in the ink mixture. The discoloration could be ascribed to a degradation path involving photoreduction of the excited dye cation to a colorless leuco form, as reported by several authors, which may occur in parallel with the described N-demethylation process (Gorshova et al., 2016).

## Conclusion

Black and blue writing inks deposited on paper were investigated by Raman and SERS spectroscopies in order to identify major dye components. SERS measurements were performed *in situ* by deposition of a small droplet of plasmonic nanopaste on the analytical surface. Blue and black Bic and Staedler inks treated with Ag nanopastes gave SERS spectra of highly enhanced intensity compared to Raman spectra and SERS spectra taken at 514 nm illumination wavelength with Au nanopastes. The large enhancement was ascribed to a combination of EM, CT, and MR effects. SERS was also used to investigate the degradation process of blue writing inks through application of artificial aging processes involving light and high temperature exposure. Interestingly, in spite of the similar initial blue coloration, it was found that aged colored paper displayed very different colorations from each other. Specifically, the Bic paper displayed a darker blue color, the Staedler-colored paper displayed almost complete discoloration and the Pilot paper showed very small color changes. This was ascribed to the different chemical composition of the constituting inks, characterized by presence of phthalocyanine and triarylmethane dye in the case of Bic pen and only triarylmethane in the case of the Staedler pen. A degradation process involving N-demethylation of triarylmethane components was proposed based on spectral evidence. However, the intense discoloration observed for Staedler pen also suggested the concomitant formation of colorless leuco forms, due to photoreduction processes.

## Data Availability Statement

All datasets generated for this study are included in the article/Supplementary Material.

## Author Contributions

DS and AA performed the Raman, SERS measurements, SEMs, and synthesis of Ag nanopastes. MT and RG performed the aging of samples. AM provided the pens and set up the aging protocols. DI and PB wrote the manuscript.

### Conflict of Interest

The authors declare that the research was conducted in the absence of any commercial or financial relationships that could be construed as a potential conflict of interest.
