# Supplementary material for: Raman Spectroscopy and Surface Enhanced Raman Scattering (SERS) for the Analysis of Blue and Black Writing Inks: Identification of Dye Content and Degradation Processes
Source: Front Chem. 2019 Oct 25;7:727. doi: 10.3389/fchem.2019.00727 (PMC6823613; doi:10.3389/fchem.2019.00727)
Supplement: Supplementary file 1 [file Presentation_1.pdf]

Supporting Information

Raman Spectroscopy and Surface Enhanced Raman Scattering (SERS) for the Analysis of Blue and Black Writing Inks: Identification of Dye Content and Degradation Processes

Daniela Saviello,[a] Maddalena Trabace,[b] Abeer Alyami,[a] Antonio Mirabile,[c] Piero Baglioni,[b] Rodorico Giorgi,[b] Daniela Iacopino[a]*

[a]Tyndall National Institute, University College Cork, Nanotechnology group, Dyke Parade, Cork, Ireland

[b]Department of Chemistry & CSGI, University of Florence, Italy

[c]Mirabile, 11 Rue de Bellefond, 75009 Paris 09, France

Corresponding author email: daniela.iacopino@tyndall.ie

Details of analyzed commercial inks

Figure S1a shows a table with detailed names and colors of the eight commercial pen inks analyzed in this work. Figure 1b-f show white light optical microscopy images of pen lines drawn on commercial Fabriano paper with some of the analyzed pens. All images show blue and black coloration arising from deep pen ink penetration into the paper fibers.

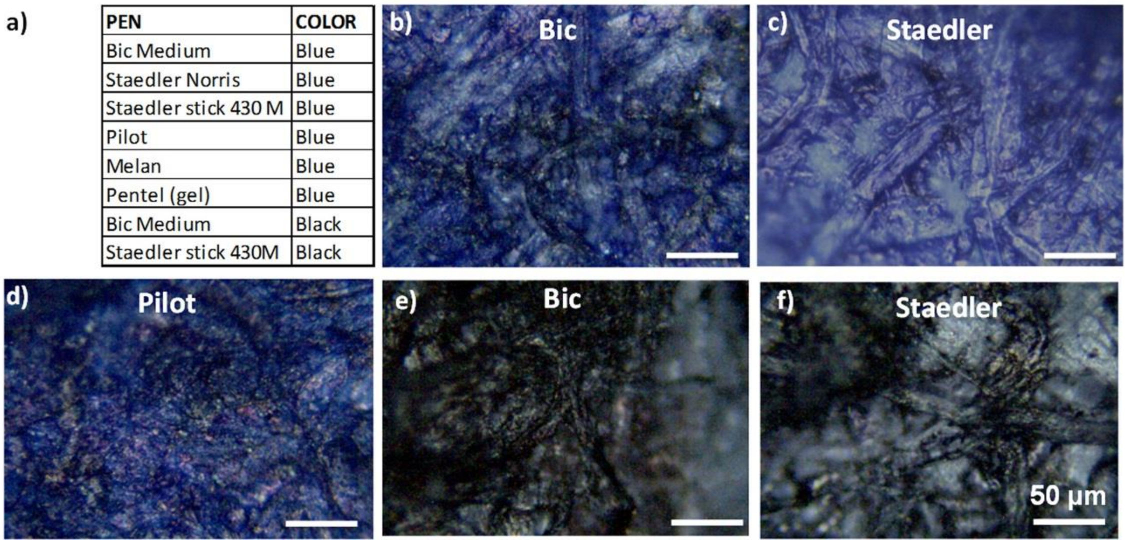

Figure S1: a) List of writing inks analyzed in this work. White light optical microscopy images of ink strokes on Fabriano paper realized with the following pens: a) blue Bic, b) blue Staedler Norris, c) blue Pilot, d) black Bic and f) black Staedler Norris. Scale bar 50 μm for all optical images.

Raman and SERS spectra of blank paper

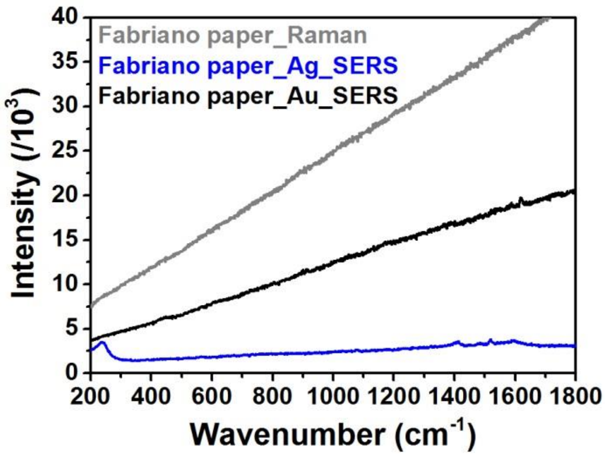

Figure S2. Raman and SERS spectra of commercial Fabriano paper

SERS analysis of analyzed writing inks

Table S1. List of band positions found in SERS spectra of analyzed inks.

SERS Ag nanopaste (cm^{-1})					SERS Au nanopaste (cm^{-1})				
Blue Bic	Blue Staedler	Blue Pilot	Black Bic	Black Stadler	Blue Bic	Blue Staedler	Blue Pilot	Black Bic	Black Stadler
1620	1620	1614	1620	1620	1618	1622	1614	1619	1623
1588	1587	1583	1589	1588	1587	1591	1583	1589	1586
1534	1536	1528	1534	1536	1536	1387	1528	1537	1538
1482	1479	1359	1478	1479	1389	1367	1359	1391	1390
1440	1446	1168	1443	1443	1367	1173	1168	1177	1372
1392	1389	1105	1388	1388	1298	913	1105	1139	1295
1371	1371	936	1370	1373	1169		936	912	1179
1294	1294	912	1298	1295	914		912	439	912
1175	1180	756	1179	1179	803		756	423	805
943	941	595	989	986	724		595		442
916	911	438	941	942	441		438		422
806	809	419	915	912	425		419		
760	759		806	810					
727	730		763	763					
565	572		733	731					
526	529		566	569					
443	444		526	526					
421	420		441	442					
337	339		421	420					
			341	342					

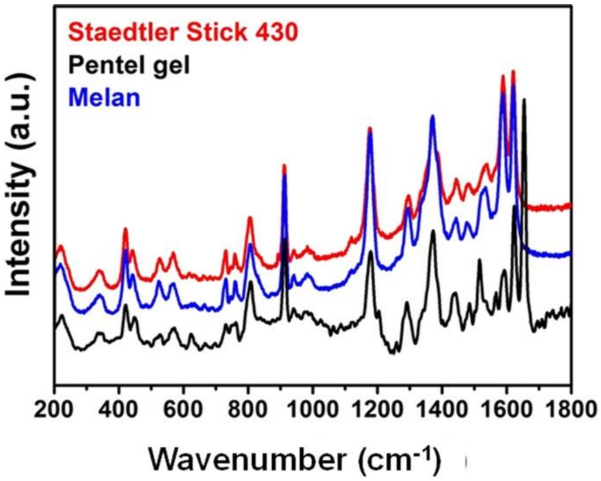

Figure S3. SERS spectra of blue pens: Staedler stick 430, Melan and gel pen Pentan obtained with 514 nm illumination wavelength.

Figure S3 shows SERS spectra of other analyzed pen inks obtained by deposition of Ag nanopaste on ink-colored paper. Staedler stick 430 and Melan pens showed nearly overlapping spectra with peaks centered at 1621, 1587, 1532, 1489, 1442, 1369, 1293, 1178,

911, 804, 759, 728 570, 523, 441 and 419 cm^{-1}, close to the peaks obtained for Staedler Norris pen shown in Figure 3 on the main manuscript. This suggests a chemical composition with presence of triarlymethane dyes. In contrast, peaks in different locations were observed for the Pentel gel pen, especially above 1400 cm^{-1} with bands centered at 1652, 1624, 1590, 1566 and 1514 cm^{-1} revealing presence of Euroglaucine and a red component, likely Rodhamine B.

SERS spectra of analyzed writing inks at 785 nm illumination wavelength

Writing inks were also analyzed with 785 nm illumination wavelength in order to investigate possible components not Raman active at 514 nm. SERS effect was obtained by deposition of Au nanopaste on the colored paper line. The SERS spectrum of blue Bic writing ink (Figure S4a) showed bands at 1537, 1338 and 719 cm^{-1} all attributable to phthalocyanine dye. The SERS spectrum of Staedler ink showed bands diagnostic of tryarylmethene dyes at 1619, 1595, 1374, 1180 and 916 cm^{-1}. In order to get more insights into the chemical composition of blue Pilot pen SERS spectra were also measured with Au nanopaste at 514 and 785 nm illumination wavelengths (Figure S4c). Bands of different intensities were obtained with higher intensity spectra recorded at 514 nm compared to 785 nm suggesting that a molecular resonance effect (SERRS) was into play at 514 nm and had a stronger effect than the plasmonic effect into play at 785 nm. The absence of additional bands at 785 nm suggests lack on phthalocyanine dyes or pigments in the chemical composition of this ink. Formulas of identified dyes are reported in Figure S5 for reference.

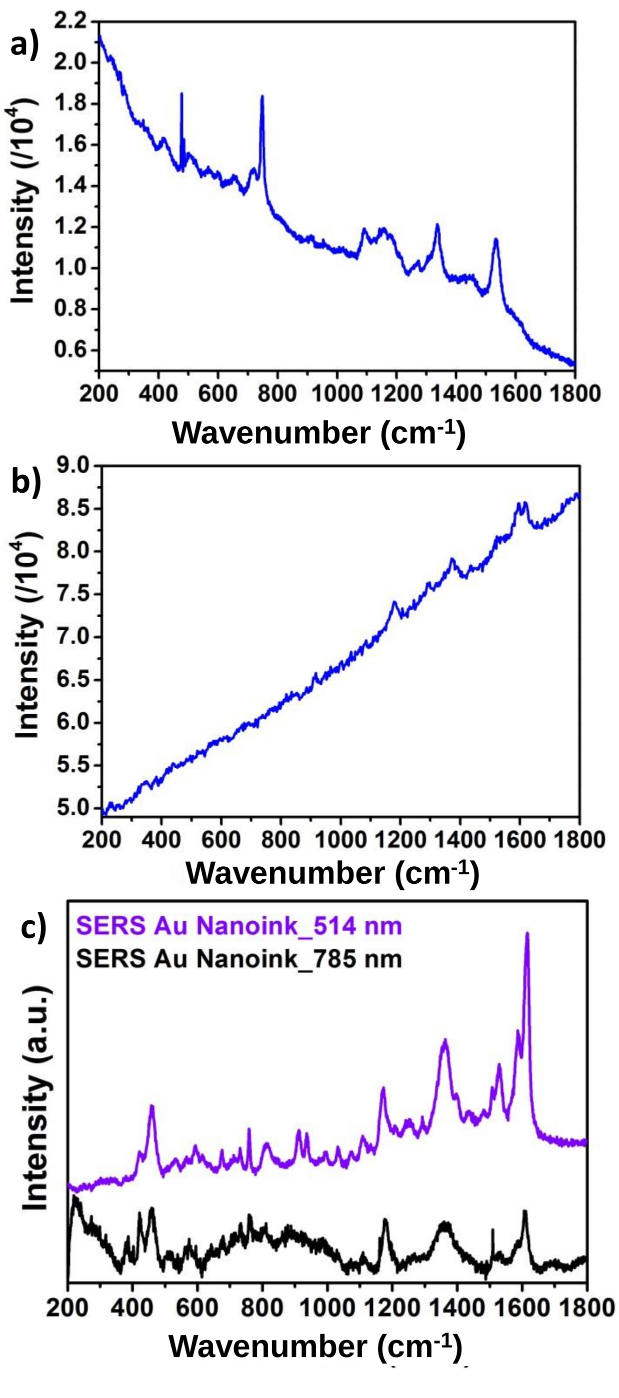

Figure S4. SERS spectra of blue writing inks obtained with Au nanopaste: a) Bic, b),Staedler, c) Pilot pen obtained with illumination at 514 nm (purple line) and 785 nm (black line).

a)

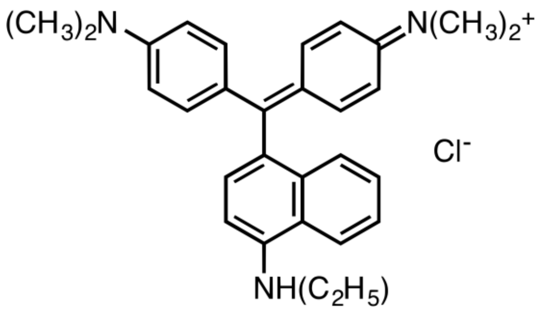

Victoria Blue R

b)

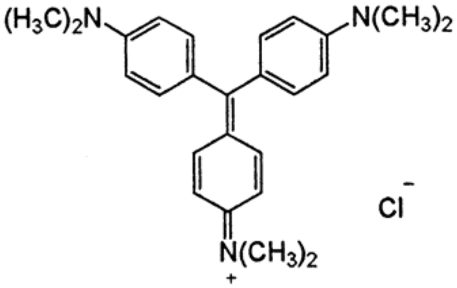

Crystal Violet

c)

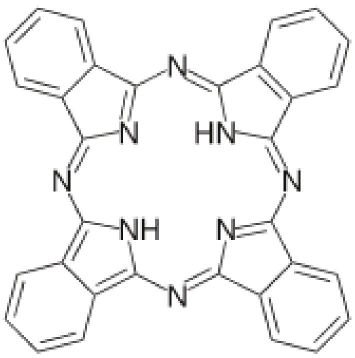

Phthalocyanine

Figure S5. Chemical structure of dyes identified in investigated writing ink mixtures.

Thin Layer Chromatography (TLC) analysis

The separation of the dye components in blue writing inks was achieved by TLC. By using an ethylacetate-ethanol-water eluent (70:35:30 v/v) blue and purple spots formed for Bic and Staedler pens whereas only blue spots formed for the Pilot pen. Purple spots of Bic and Staedler pens showed retention factors (Rf) equal to 0.51. Blue spots with higher Rf showed in the TLC of Staedler, suggesting the presence of additional blue dyes in the ink mixture.

Pilot pen main blue spot showed an Rf of 0.37. For comparison, reference dye Crystal Violet was deposited on another TLC which was run in parallel with the ink TLC.

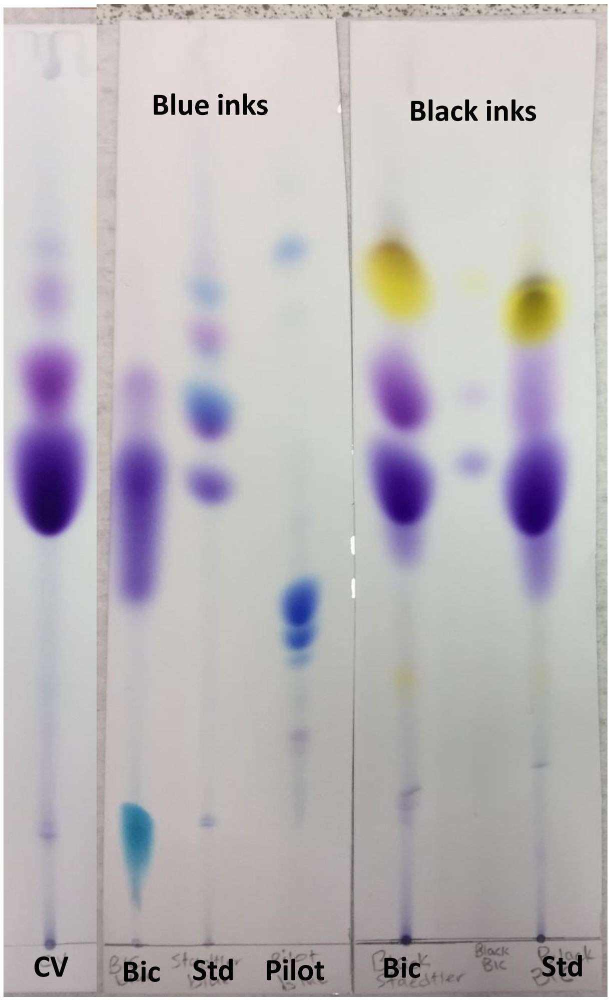

Figure S6. Photograph of three TLC plates showing blue Bic, Staedler and Pilot inks and black Bic and Stadler inks against reference dye Crystal Violet.

Deposited CV displayed Rf equal to = 0.51, equivalent to the Rf of the Bic and Staedler purple spots, respectively. Purple spots in black Bic and Staedler inks also showed Rf = 0.51, corresponding to the presence of CV. Unidentified Yellow spots also formed at higher Rf = 0.71.
